# Prescribing Patterns of Antihypertensive Medications in US Ambulatory Care Settings

**DOI:** 10.3390/pharmacy7020064

**Published:** 2019-06-14

**Authors:** Yelena Sahakian, Brisilda Bylykbashi, Ateequr Rahman

**Affiliations:** College of Pharmacy, Rosalind Franklin University of Medicine and Science, North Chicago, IL 60064, USA; yelena.sahakian@my.rfums.org (Y.S.); brisilda.bylykbashi@my.rfums.org (B.B.)

**Keywords:** prescribing pattern, adherence, hypertension

## Abstract

Over 70 million Americans are diagnosed with hypertension. Adherence to current AHA/ACC 2017 hypertension guidelines and appropriate antihypertensive therapy is important for optimal treatment outcomes. This study investigates prescribing patterns for ambulatory care patients with hypertension and adherence to these guidelines. Data from the 2015 National Ambulatory Medical Care Survey (NAMCS) were used in the study. Patients with primary diagnoses of essential hypertension were extracted from the data using ICD-9 code “401”. A total of 595 patients were identified. Correlation among demographic variables, source of payment and prescriber specialty were examined. Chi-square and descriptive analysis were performed. 51.4% of the prescriptions were non-first-line medications. Primary care physicians and cardiologists adhered to the guidelines more, when compared to the other specialties. There was a significant difference between various geographic regions, as it relates to guidelines adherence. This study concluded that prescribers do not always adhere to the AHA/ACC 2017 hypertension guidelines. It is recommended to adhere to the guidelines if there are no contraindications. The study’s findings were limited to the ambulatory patients visiting providers in 2015 and by the operational definitions of the study.

## 1. Introduction

About 74 million Americans, or one in three adults, have high blood pressure. Hypertension is a silent disease that does not show symptoms until it has caused permanent damage to the heart, brain, kidneys or eyes [1,2]. Unfortunately, about a quarter of people with high blood pressure do not know they have the condition. Half of the people being treated for the disease gradually increase the dose to reach the target blood pressure, which is 130/80 mmHg for patients with no clinical Cardiovascular Disease CVD and having a less than 10% 10-year Atherosclerotic Cardiovascular Disease (ASCVD) risk, and 130/80 mmHg for patients with diabetes, chronic kidney disease, or any type of atherosclerosis, such as peripheral artery disease, aortic aneurysm, and coronary artery disease [3,4].

Control of hypertension is one of the most important health care priorities and is expected to increase with an aging population and increasing burden of obesity and other risk factors related to lifestyle. Hypertension is primarily managed in ambulatory settings accounting for an estimated 79% of primary care visits [5]. Better hypertension control can positively impact cardiovascular health [6,7]. Recent studies have identified increased use of antihypertensive agents coincides with increased control over blood pressure both in younger and older populations and decreased cardiovascular mortality, myocardial infarction and stroke rates [8,9,10].

Appropriate antihypertensive therapy in outpatient care is important for optimal treatment outcomes. According to recent guidelines, there are different blood pressure goals using an age cutoff of 50 years. National Ambulatory Medical Care Survey (NAMCS) data analysis for the years 2003–2010 revealed antihypertensive medication prescription increased from 69.2% in 2003–2004 to 78.8% in 2009–2010 (P = 0.001) with increased blood pressure control from 39.1% to 48.8% (P < 0.001). The trend was consistent in patients younger, as well as older than 60. Prescription for beta-blocker has increased from 25.4% to 34.7% while ARB prescription increased 17% to 22.1. African American patients, those with other comorbidities, insufficient insurance coverage and younger patients are less likely to control their blood pressure. Adults aged 18–39 have the lowest hypertension control rates among adults with hypertension in the US [11]. Surprisingly, older patients are more likely to control their blood pressure than the younger population, which may be due to lack of disease awareness and management or resistance to initiation and escalation of antihypertensive therapy [12]. 

The goal of this study is to determine prescribing pattern for patients with hypertension for the year of 2015 and determine if they are being prescribed first-line therapy and evaluate the adherence of prescribers to current guidelines. 

Non-adherence to first-line therapy is one of the drivers for economic implications as it may lead to treatment failure, disease progression and more complex treatments, resulting in increased cost of treatment. According to AHA, the estimated direct and indirect cost of hypertension in the US was 76.6 billion dollars in 2010 [13]. The direct medical costs of CV disease may be substantially higher than this estimate since it includes secondary hospitalizations, which accounts for 18,953 dollars per patient annually as direct medical costs. Additionally, patients with secondary hospitalizations coat 4.5 times higher than those with no hospitalizations [14].

## 2. Materials and Methods 

Data from 2015 NAMCS were used in the study. Patients data with primary diagnoses of essential hypertension were extracted by using ICD-9 code “401”. The National Ambulatory Medical Care Survey (NAMCS) is designed to meet the need for objective, reliable information about the provision and use of ambulatory medical care services in the United States. 

A total of 595 patients were found which represented the study sample. Patient age, sex, race, as well as the source of payment, and the provider type were among the operational factors used in data analysis. Sources of payment were defined as coverage through either private insurance, Medicaid, Medicare, or other state-based programs, self-pay, work compensation, and charity. All variables were categorized as “patient” or “physician” factors. Geographic region was the only variable categorized as both patient and physician.

Data analysis was performed using SPSS software for descriptive data analyses and statistical coding. Various demographic variables were analyzed using frequencies, means and other descriptive statistics. The data was further analyzed using chi-square to compare proportions between groups. The extracted data set was checked for integrity, equality and distribution of a number of records in every phase of analysis. The hypothesis focused on physician variables to determine whether the number of non-first-line antihypertensive agents prescribed was related to physician’s specialty, geographic region and other demographic variables, such as age, gender, race and payment type. The definition of prescribing appropriateness was determined by 2017 AHA/ACC guidelines. Thiazides, ACE inhibitors, ARBs and CCBs were defined as first-line and appropriate treatment agents, whereas loop diuretics, beta-blockers, direct renin inhibitor, alpha-1 blockers, direct vasodilators, central alpha-1 agonists and other centrally acting drugs were defined as non-first line agents. 

## 3. Results

Females and white patients represented over half of the sample. Patients aged 65 and older represented half of the sample, followed by those aged 45. Majority of the patients had Medicare as primary insurance (64.45%), and 39.3% were private insurance holders. General doctors (34.8%), cardiovascular specialists (32.1%) and internal medicine physicians (21.8%) were the majority. Most of the visits occurred in the South (36.5%) and Midwest (26.9%) (Table 1). After analyzing the data, 51.4% of prescriptions turned out to be non-first-line prescriptions. 

Female patients (X^2^ = 0.376) and those aged 65 and older (X^2^ = 0.073) received more non-first-line agents. White, African American patients (X^2^ = 0.208), as well as Medicare and private insurance holders (X^2^ = 0.145) were prescribed more non-first-line antihypertensive agents. Chi-square analysis showed that South and Midwest received more non-adherent prescriptions (X^2^ = 0.023). Cardiovascular specialists, family doctors and internal medicine specialists prescribed more non-first-line agents (X^2^ = 0.222) (Table 2).

## 4. Discussion

This study reveals not only significant differences in antihypertensive drug utilization among various demographics and physician specialties but also lack of adherence to the prescribing guidelines. Female and white patients, representing the majority of the sample, were prescribed more non-first-line treatments, as was the case with patients 65 and older. Since this population represented half of the sample, they also represented the subgroup having Medicare. South and West had more cases of non-first-line prescribing pattern due to being the majority (X^2^ = 0.023). This corresponds to data from other studies about the South being one of the geographical areas with poor health and wellness. 

Primary (essential) hypertension is diagnosed in the absence of an identifiable secondary cause. It is characterized by severe headache, fatigue or confusion, vision problems, difficulty breathing among other symptoms. The rise in antihypertensive drug utilization represents a potentially favorable development in terms of increased numbers of patients undergoing drug treatment. 

Cardiovascular specialists and general doctors who were the most common provider seen by the patients, were also one of the provider specialties prescribing the majority of non-first-line agents, which corresponds to hypertension being a cardiovascular condition. 

The data analysis showed that half of the antihypertensive prescriptions are not first-line agents, which can imply less effective drug therapy and worse clinical outcomes. This is where the issue of medication non-adherence comes into play to ensure the effectiveness of first-line therapy if there are no contraindications, to avoid side effects associated with non-first-line agents, such as beta-blockers and loop diuretics and to reach a better blood pressure control. 

Over 25% of people prescribed antihypertensive medications stop taking them 6 months after initiation of therapy. Medication adherence is influenced by physician communication and medical management skills, even though they can be improved by educational and practice interventions [15]. The risk of non-adherence can be reduced with better medical management, communication skills, early therapy changes, more follow-up visits, and lastly, starting therapy with nondiuretic agents. A study by Tamblyn et al. found that medical management can reduce non-adherence by 15.8% [16]. Understanding barriers in hypertension management will inform the development of interventions to control blood pressure in the young adult population. Such barriers include altered self-identity, greater blood pressure variability, possible side effects initiating a medication, gender differences among young adults were also barriers to follow-up and new hypertension medication initiation. The pressure of having an effective motivational interview with productive outcomes, cost of the medication and the lack of motivation from the patient to schedule a follow-up visit can make hypertension management even harder. Thus, taking measures directed to young adults are essential to improve hypertension care in this population [11].

Conn et al. suggest that habit-focused interventions reduce blood pressure more effectively because they examine participants’ daily routines and incorporate medication administration to routine events. Such interventions improve clinical outcomes by improving adherence. Habit-based interventions can also be tailored into a patient’s daily routine to enhance a healthy lifestyle, which in turn will positively impact blood pressure control. Interventions delivered by pharmacists were found to be most effective, as patients see them as experts in medication therapy and are very receptive to their recommendations [17]. 

The study was limited by the operational definitions of the study variables and the data collection period. Another limitation was the number of ambulatory care visits during the year of 2015. The variable selection was based on the available NAMCS database. In addition, the patients were assumed to have an initial diagnosis of hypertension, rather than a recurrent visit. 

Another limitation of the study was that the 2015 guidelines include beta-blockers along with ACE inhibitors or ARBs, and thiazide or thiazide-like diuretics in the first-line treatment in those with a history of MI. The 2017 ACC/AHA guidelines did not indicate beta-blockers as first-line treatments. Although the analysis criteria for this study were for essential hypertension, there may have been few patients who were prescribed only a beta-blocker and were considered as non-first line therapy. No allergy information was available, and the patients not given the first-line agent because of the allergy were not identified. Lastly, treatments consisted of agents other than ACEI, thiazides, CCBs, ARB or a combination of any of the above agents along with the treatments with a primary diagnosis of hypertension and not receiving any therapy were considered non-first-line. 

Large differences in the utilization of different groups of antihypertensive agents were noted in this study. Underutilization of valuable, efficacious, and cost-effective thiazide diuretics and overuse of expensive ACE inhibitors is unjustifiable. Therefore, more attention should be paid to their implementation in order to improve health gains. Clinicians may use these findings to tailor antihypertensive therapy to the needs of each patient. 

## Figures and Tables

**Table 1:** Demographics.

Demographic Variables	Frequency (n)	Percentage
Gender		
Female	324	54.5
Male	271	45.5
Age Range		
15–44 years	50	8.4
45–64 years	245	41.2
65–74 years	156	26.2
75 and older	144	24.2
Race		
White	347	58.3
African American	124	20.8
Other	21	3.5
Blank	103	17.3
Type of Payment		
Private Insurance	234	39.3
Medicare	268	45
Medicaid	38	6.4
Other	55	9.3
Physician Specialty		
General/Family practice	207	34.8
Internal medicine	130	21.8
Cardiovascular diseases	191	32.1
Physician assistant	15	2.5
Nurse practitioner	8	1.3
Other specialties	44	7.4
Region		
Northeast	102	17.1
Midwest	160	26.9
South	217	36.5
West	116	19.5

**Table 2:** Prescribing patterns.

Prescribing Pattern Based on Demographic Variables	First-Line Therapy (n) (%)	Non-First-Line Therapy (n) (%)	Significance
			(P)
Percentage	48.60%	51.40%	
Gender			0.376
Female	172 (28.9%)	152 (25.5%)	
Male	134 (22.5%)	137 (23.0%)	
Age			0.073
15–44	20 (3.3%)	30 (5.0%)	
45–64	130 (21.8%)	115 (19.3%)	
65–74	78 (13.1%)	78 (13.1%)	
75 and older	78 (13.1%)	66 (11.0%)	
Race			0.208
White	175 (29.4%)	172 (28.9%)	
African American	70 (11.7%)	54 (9.0%)	
Other	61(10.2%)	63 (10.5%)	
Insurance type			0.145
Private Insurance	119 (20.0%)	115 (19.3%)	
Medicare	145 (24.3%)	123 (20.6%)	
Medicaid	15 (2.5%)	23 (3.8%)	
Other	27 (4.5%)	28 (4.7%)	
Region			0.023
Northwest	55 (9.2%)	47 (7.8%)	
Midwest	90 (15.1%)	70 (11.7%)	
South	116 (19.4%)	101 (11.6%)	
West	45 (7.5%)	71 (11.9%)	
Physician			0.222
General/Family practice	95 (15.9%)	112 (18.8%)	
Internal medicine	65 (10.9%)	65 (10.9%)	
Cardiovascular diseases	112 (18.8%)	79 (13.2%)	
Other specialties	33 (5.5%)	34(5.6%)	
